# Dual ultrasound-guided totally implantable venous access ports via the right internal jugular vein in pediatric patients with cancer: a preliminary experience in a single institution

**DOI:** 10.1136/wjps-2022-000509

**Published:** 2023-06-29

**Authors:** Xiao Bin Deng, Liang Peng, Jun Zhang, Xiangru Kong, Zhenzhen Zhao, Shan Wang, Changchun Li, Yifei Du, Jianwu Zhou, Lifei Liu, Chao Yang

**Affiliations:** 1 Department of Surgical Oncology, National Clinical Research Center for Child Health and Disorders, Ministry of Education Key Laboratory of Child Development and Disorders, China International Science and Technology Cooperation Base of Child Development and Critical Disorders, Chongqing Key Laboratory of Pediatrics,Chongqing Medical University Affiliated Children's Hospital, Chongqing, China; 2 Department of Pediatric Anesthesia, National Clinical Research Center for Child Health and Disorders, Ministry of Education Key Laboratory of Child Development and Disorders, China International Science and Technology Cooperation Base of Child Development and Critical Disorders, Chongging Key Laboratory of Pediatrics,Chongqing Medical University Affiliated Children's Hospital, Chongqing, China

**Keywords:** Pediatrics, Hospitals, Pediatric

## Abstract

**Objective:**

To assess the efficacy and safety of dual ultrasound-guided (DUG) totally implantable venous access port (TIVAP) implantation (namely, using ultrasound-guided percutaneous puncture with transesophageal echocardiography-guided catheterization) via the right internal jugular vein (IJV) in pediatric patients with cancer.

**Methods:**

Fifty-five children with cancer requiring chemotherapy underwent DUG-TIVAP implantation via the right IJV. Clinical data were recorded, including the procedure success rate, first attempt success rate, and perioperative and postoperative complications.

**Results:**

All 55 cases were successfully operated on. The first puncture success rate was 100%. The operation time was 22–41 min, with a mean time of 30.8±5.5 min. The mean TIVAP implantation time was 253±145 days (range 42–520 days). There were no perioperative complications. The postoperative complication rate was 5.4% (3/55), including skin infections around the port in one case, catheter-related infection in one case, and fibrin sheath formation in one case. The ports were all preserved after anti-infection or thrombolytic therapy. No unplanned port withdrawal was recorded in this study.

**Conclusions:**

DUG-TIVAP implantation is a technique with a high success rate and a low complication rate; therefore, it provides an alternative for children with cancer. Further randomized controlled studies are needed to confirm the efficacy and safety of DUG-TIVAP via the right IJV in children.

**WHAT IS ALREADY KNOWN ON THIS TOPIC**

- There are no reports on dual ultrasound-guided (DUG) totally implantable venous access port (TIVAP) implantation.

**WHAT THIS STUDY ADDS**

- DUG-TIVAP implantation provides a new method for implantable venous access port implantation.

**HOW THIS STUDY MIGHT AFFECT RESEARCH, PRACTICE OR POLICY**

- DUG-TIVAP can reduce X-ray exposure in patients. This study shows that it can improve the puncture success rate and reduce complications.

## Introduction

Long-term central venous access has become essential for patient treatment, including pediatric patients with cancer undergoing chemotherapy, total parenteral nutrition, and frequent blood sampling.^1^ Totally implantable venous access ports (TIVAPs) and peripherally inserted central catheters (PICCs) are the most commonly used venous access routes. Compared with PICCs, TIVAPs have become more widely used in recent years as they are easier to care for and are associated with lower rates of complications.^2^ The internal jugular vein (IJV) approach is the most commonly used method. Alternative approaches include the subclavian (SCV), cephalic, and innominate veins. Currently, ultrasound and fluoroscopic guidance are used frequently for TIVAP placement because this approach is associated with low rates of periprocedural and postoperative complications.^3^ However, multiple intraoperative X-ray examinations are required for fluoroscopic guidance, and there is a certain degree of radiation exposure. Here, we report a new approach for TIVAP placement using percutaneous ultrasound-guided and transesophageal echocardiography (TEE)-guided (which we named dual ultrasound-guided (DUG)) catheterization in pediatric patients with cancer, and we evaluate the efficacy and safety of DUG-TIVAP implantation.

## Methods

### Patients

Clinical and nursing data were collected from 55 children with cancer who underwent DUG-TIVAP implantations via the right IJV from January 2019 to April 2020. The TIVAP used in this study was purchased from B. Braun Medical (Celsite, 5 F epoxy resin catheter, port weight 3 g, REF 04433734). All patients underwent surgery performed by the same surgical group.

### Implantation procedures

All surgeries were performed in an operation room under general anesthesia via an inhalation cannula. The right IJV was our preferred catheter access site. If there were skin erosions or hematomas at or near the insertion site or if thrombosis or small-diameter veins were detected by preoperative ultrasound, the left IJV was chosen. These patients were excluded from the study. Patients with any contraindication to TEE, including severe arrhythmia, severe heart failure, esophageal stricture, ulcer, perforation, bleeding or local hematoma, or a history of esophageal surgery or mediastinal radiotherapy, were excluded. Patients undergoing SCV vein punctures for other indications were excluded.

**Figure 1:**
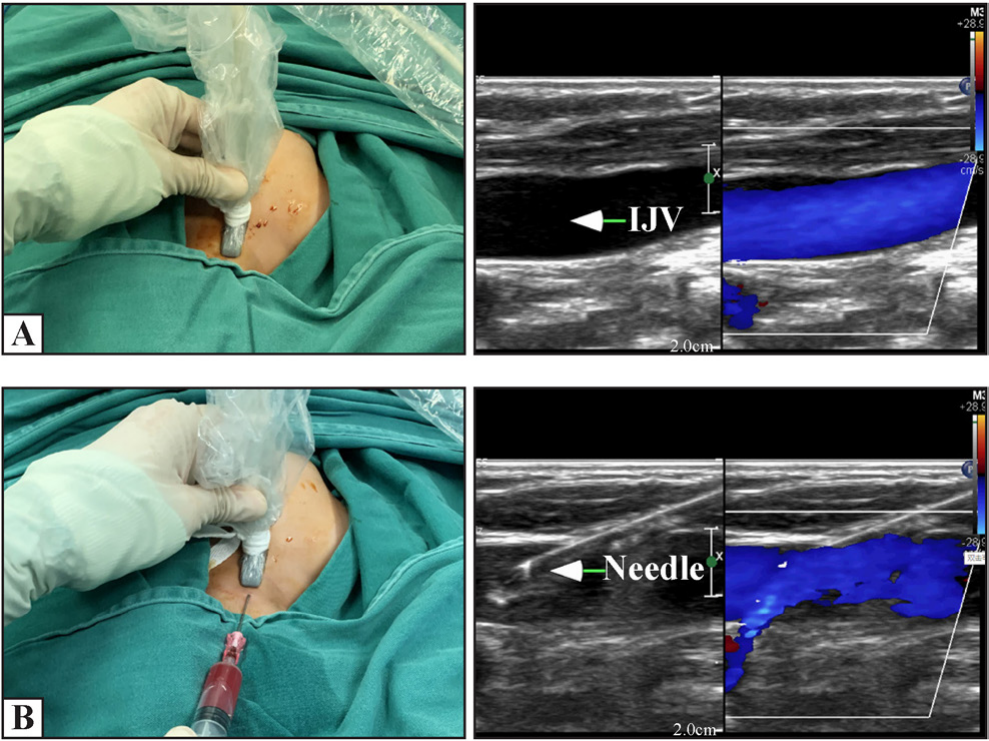
Long-axis in-plane ultrasound-guided IJV puncture. (A) The long axis of the IJV is displayed in the ultrasound image. (B) The needle passes through the anterior wall of the vessel, and the blood can be aspirated. IJV, internal jugular vein.

**Figure 2:**
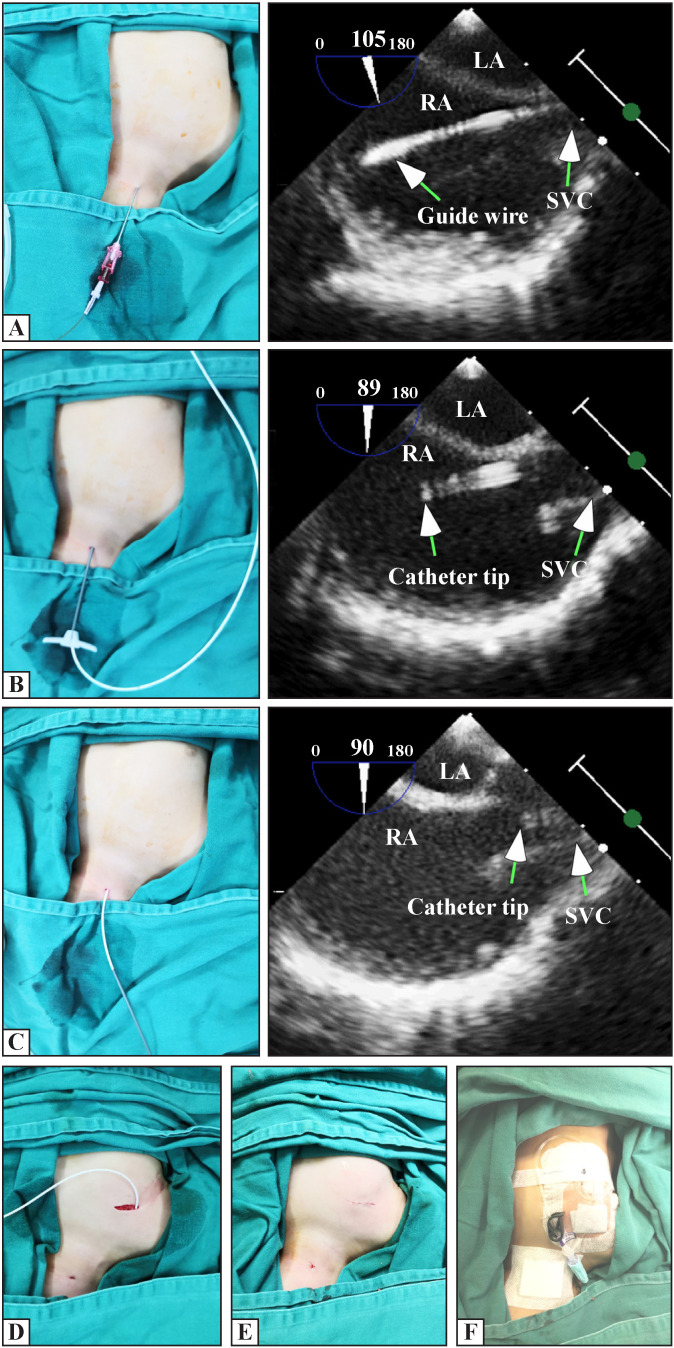
Transesophageal echocardiography-guided port catheter implantation. (A) The guide wire was in the RA. (B) The port catheter was inserted into the RA. (C) The catheter tip was placed at the level of the cavoatrial junction. (D) A port catheter was drawn, crossing above the clavicle using a tunnel needle. (E) The skin incision was closed by continuous intracutaneous suture. (F) Picture at the end of the operation. LA, left atrium, RA, right atrium; SVC, superior vena cava.

After anesthesia, the patients lay supine with their shoulders raised and heads turned to the left. After skin disinfection and sterile draping, the percutaneous ultrasound-guided Seldinger technique^4^ was performed. First, the right IJV was punctured under ultrasound guidance using the Affiniti 50 Diagnostic Ultrasound system (Philips, Bothell, Washington, USA) with a high-frequency 23 mm broadband linear array probe (7–15 MHz) using the long-axis in-plane technique (figure 1A,B). After a successful puncture, a guide wire with a J-shaped tip was inserted. Then, we used the Affiniti 50 Diagnostic Ultrasound system (Philips) with a s7-3t TEE probe (3–7 MHz) to confirm that the guide wire was in the superior vena cava or right atrium (figure 2A). Next, we made a 0.2 cm incision at the puncture point, followed by introducer sheath insertion. Subsequently, the port catheter was inserted into the superior vena cava along the catheter sheath (figure 2B). The aim was to place the catheter tip at the level of the cavoatrial junction. It was calculated according to the formula [length of inserted catheter=height/10−1 cm (height <100 cm) or −2 (height>100 cm)].^5^ This was then confirmed and adjusted using TEE (figure 2C). The calculated value and TEE confirmed value were both recorded. We also analyzed the consistency rate of the length calculated by the formula and the length after localization by transesophageal ultrasound at the final surgery. Next, a subcutaneous pocket was prepared on the anterior thoracic wall, followed by a tunnel needle to draw the port catheter crossing above the clavicle (figure 2D). The catheter was cut to a suitable length connected to the port. The port was fixed using two absorbable sutures (4–0 coated Vicryl; Ethicon, Germany). Then, the function of the port was verified using a percutaneous aspiration injection with a non-coring needle, followed by a 10 U/mL heparin infusion. Finally, the skin incision was closed using continuous intracutaneous sutures (5–0 coated Vicryl, Ethicon) (figure 2E), and covered with Tegaderm IV transparent dressings(3M, USA) (Figure 2F). Every skin puncture was defined as an attempt. The puncture time was defined as the time from the skin puncture to the detection of the guide wire entering the right atrium using TEE.

The nurses who maintained and managed the TIVAPs were professionally trained according to the consensus of Chinese experts^6^ and American guidelines for the prevention of catheter-related infections.^7^ Perioperative and postoperative complications were defined and recorded according to the reporting standards of the Society of Interventional Radiology.^8–10^ Perioperative complications were defined as complications occurring during the implantation surgery or within the first 24 hours after implantation and included hematoma, misinsertion into an artery, pneumothorax, and hemothorax. Hematoma was defined as visible or palpable swelling around the puncture site. Misinsertion into an artery was defined as high pressure and red arterial blood flow out of the needle, with it visible inside the artery on ultrasound. Pneumothorax was defined as the disappearance of pulmonary sliding and comet-tail artifacts on ultrasound. Hemothorax was described as a hypoechoic area detected above the diaphragm and inside the chest wall by ultrasound.^11^ Early complications were defined as complications occurring within 24 hours to 2 weeks after catheter implantation. Late complications were defined as those that occurred beyond the second week after implantation.

## Results

The patient characteristics are shown in table 1. There were 23 patients who were newly diagnosed; the median absolute neutrophil count (ANC) was 5×10^9^/L (range from 1.3 to 25×10^9^/L), and the median platelet count was 180×10^9^/L (range from 86 to 827×10^9^/L). The other 32 patients underwent operation after chemotherapy; the median ANC of these patients was 1.2×10^9^/L (range from 0.5 to 7.2×10^9^/L), and the platelet count was 105×10^9^/L (range from 51 to 568×10^9^/L).

**Table 1:** Patient characteristics

Characteristics	Values (n=55)
Age (years)*	2.72±2.43 (0.25–7.5)
Female, n (%)	25 (45.5)
Body weight (kg)*	13.2±5.8 (6–30)
Height (cm)*	89.9±19.5 (60–140)
Cancer types, n (%)	
Leukemia	17 (30.9)
Neuroblastoma	13 (23.6)
Hepatoblastoma	7 (12.7)
Rhabdomyosarcoma	6 (10.9)
Lymphoma	5 (9.1)
Germ cell tumor	5 (9.1)
Wilms’ tumor	2 (3.7)

*Data are presented with mean±SD (range).

The operation was successful in 55 cases (table 2). The success rate for the first puncture was 100%. The operation time was 22–41 min, with a mean time of 30.8±5.5 min. The calculated length of the catheter was consistent with the TEE-confirmed value in 83.7% (46/55) of patients. The length consistency rate was 66.7% (8/12) and 88.4% (38/43) in children younger than 1 year and older than 1 year, respectively. The calculated value was 0.5–1.0 cm longer or shorter than the TEE-confirmed value in infants. The mean TIVAP time was 253±145 days (range 42–520 days).

**Table 2:** Details of dual ultrasound-guided TIVAP via the right IJV

Details	Values (n=55)
Success rate of first attempt, n (%)	55 (100)
Success rate of surgery, n (%)	55 (100)
Misinsertion of artery, n (%)	0 (0)
Times of puncture (min)*	1.1±0.7 (0.3-2.0)
Operation time (min)*	30.8±5.5 (22–41)
Length of catheter introduction (cm)*	8.5±1.8 (6.5–13.5)
Length consistent rate, n (%)	
<1 year (n=12)	8 (66.7)
>1 year (n=43)	38 (88.4)
TIVAP time (days)	253±145 (42–520)

*Data are presented with mean±SD (mean).

IJV, internal jugular vein; TIVAP, totally implantable venous access port.

Postoperative complications were shown in table 3. No hematomas, pneumothorax or hemothorax was observed. There were no early postoperative complications. No wound infection or wound dehiscence was observed. The late complication rate was 5.4% (3/55), including skin infections around the port in one case, catheter-related infection in one case, and fibrin sheath formation in one case. The ports were preserved after anti-infection or thrombolytic therapy (table 3). No unplanned port withdrawal was recorded in this series. No catheter ectopic or catheter rupture occurred during the follow-up period.

**Table 3:** Incidence of postoperative complications and actions taken

Complications	n (%)	Actions taken
Wound infection	0 (0)	
Wound dehiscence	0 (0)	
Catheter-related infection	1 (1.8)	Antibiotics
Skin infections	1 (1.8)	Antibiotics
Fibrin formation	1 (1.8)	Thrombolysis
Total	3 (5.4)	

## Discussion

TIVAPs are widely used for the infusion of chemotherapy drugs and parenteral nutrition, significantly reducing the workload of nursing staff.^12^ Combined ultrasound-guided and fluoroscopy-guided port catheter implantation has replaced the traditional method of surgical cutdown of veins because it is a safe and reliable procedure with low periprocedural, early, and late complication rates.^3^ In this study, we developed a new implantation approach using percutaneous ultrasound-guided and TEE-guided catheterization called DUG implantation. The results showed a high technical success rate of 100%, few perioperative complications, and high levels of postoperative comfort in children. Although we did not conduct a randomized controlled trial, the operation time was reduced to 30.8±5.5 min in this study compared with our previous study,^13^ where it was 45.7±23.1 min with SCV vein catheterization and 75.9±32.4 min with IJV catheterization. Although the shorter operation time may be due to improved surgical skills, the role of dual ultrasound guidance cannot be dismissed. In contrast, the success rate of the traditional surgical venous cutdown technique is lower.^13^ The results were better when the radiological Seldinger technique was used, with a success rate ranging from 98.4% to 100%.^14 15^ The results suggest that the DUG technique might be superior to the traditional surgical technique and non-inferior to the radiological technique employed in our earlier study. Nevertheless, a prospective randomized trial comparing these methods is needed to verify this statement.

Determining the optimal venous catheter length during insertion is an issue for the surgeon, as there is currently no well-accepted strategy for ensuring correct catheter tip positioning. Fluoroscopy or chest radiography-guided port catheter implantation has been widely used to confirm tip placement. However, chest radiography has been criticized for its inadequacy in identifying the tip position^16^ and the cavoatrial junction.^17^ In addition, radiation exposure is a problem worth considering because the patient would receive at least three chest radiographs during this procedure (for guide wire confirmation, catheter tip confirmation, and post port-implantation confirmation). TEE is a more direct and accurate method to determine the position of the catheter tip, as the guide wire and catheter tip can be easily detected at the entrance of the superior vena cava. This method is more accurate than the formula, and we found that the value of the calculated length was consistent with the TEE-confirmed value in 83.7% (46/55) of patients. The inconsistency rate was higher in infants due to the tip uncertainty principle, where neck and shoulder motion and respiration can make more significant changes due to smaller body habitus. Thus, if we used the formula to calculate the catheter length and used chest radiography to ensure the catheter tip position, more radiography would be needed to adjust the optimal catheter tip position, leading to longer procedure durations and more radiation exposure.

Notably, there were no significant complications in the 55 patients. No pneumothorax or hemothorax occurred, suggesting that the DUG procedure is safe and minimally invasive even in very young patients (3 months) with low weights (6 kg). The overall complication rate in our analysis was lower than that reported by several studies.^17–19^ The most common complication was an infection, including catheter-related infection and skin infection. The port infection rate in the related literature ranges from 2.6% to 12.0%, depending on the catheter type and location and the patient’s constitution.^20 21^ There were no port removals in our study. Thromboembolism is another long-term problem of TIVAP use in patients with cancer. The thromboembolic event rate was as high as 66% in the 1990s^22^; however, the incidence has decreased dramatically in recent years. The low incidence of thrombosis may be due to specialized maintenance and management of TIVAPs according to guidelines and consensus. In addition, all patients were required to have preoperative ANC of >0.5×10^9^/L and platelet counts of >50×10^9^/L. Although some patients may experience significant decreases in ANC and platelet counts after chemotherapy, careful and standardized care during and after surgery can still reduce the incidence of postoperative wound infections and bleeding.

There are some limitations associated with the DUG procedure. Patients must be excluded if they have any contraindications to TEE. Despite the low occurrence of complications, TEE may be associated with esophageal mucosal injury and bleeding. However, no related complications were observed in the more than 4000 cases performed in our institution since the introduction of esophageal ultrasound. Esophageal ultrasound equipment is expensive and may not be available in every hospital. The technique (the precise positioning of the catheter tip) requires the participation of a professional ultrasound technician. Moreover, the detection scope of esophageal ultrasound is narrow. If the guide wire enters other veins, such as the SCV vein instead of the superior vena cava, it cannot be detected using TEE. In this case, percutaneous ultrasound might be an option. However, it may be challenging to visualize the superior vena cava by percutaneous ultrasound due to the overlying lung, and chest radiography would be needed to determine the position of the guide wire.

Electrocardiographic-guided insertion may be less invasive and has been demonstrated in many articles. However, this method has certain disadvantages, including but not limited to the fact that localization may be inaccurate,^23^ and characteristic P waves may not always be visible.^24^ An ultrasound or X-ray may still be needed.

This study aimed to reduce radiation, shorten the operation time, improve the success rate, and reduce complications. Our preliminary results suggest that we achieved these objectives. Thus, our study offers a new option for TIVAP implantation, especially for children who cannot be guided by electrocardiography (those with atrial fibrillation or a pacemaker). A more extensive comparison of this method with the conventional approach will also be carried out in the future.

In conclusion, DUG-TIVAP implantation has a high success rate and a low periprocedural complication rate; therefore, it provides an alternative for children with cancer. Although the preliminary results reported in this paper are promising, because of this study’s retrospective nature and the limited number of cases, further randomized controlled studies are needed to validate the efficacy and safety of DUG-TIVAP via the right IJV in children.

## Data Availability

All data relevant to the study are included in the article or uploaded as supplementary information.
